# Effects of IL-6 and AG490 on regulation of Stat3 signaling pathway and invasion of human pancreatic cancer cells in vitro

**DOI:** 10.1186/1756-9966-29-51

**Published:** 2010-05-19

**Authors:** Chen Huang, Guang Yang, Tao Jiang, Kejian Huang, Jun Cao, Zhengjun Qiu

**Affiliations:** 1 Department of General Surgery, Affiliated First People's Hospital, Shanghai Jiao Tong University, Shanghai, 200080, PR China; 2 Department of General Surgery, Taian central hospital, Shandong, 27100 PR China

## Abstract

**Background:**

Signal transducer and activator of transcription 3 (Stat3) is a member of the Janus-activated kinase(Jak)/Stat signaling pathway. Abnormal activation of Stat3 plays a critical role in metastasis and invasion in varieties of human tumors including pancreatic cancer. This study aimed to investigate the mechanisms of activation and blocking of the Stat3 signaling pathway and its effects on invasion and metastasis of human pancreatic cancer cells.

**Methods:**

The Jak inhibitor AG490 and interleukin-6 (IL-6) were added to the culture media of human pancreatic cancer cells SW1990 and Capan-2, respectively. Cell growth was measured by MTT assays. Western blotting and immunocytochemistry were performed to detect phosphorylated Stat3 (p-Stat3) protein, while VEGF and MMP-2 mRNA and protein expression were examined with fluorescence quantitative polymerase chain reaction and Western blotting, respectively. The invasion ability of SW1990 and Capan-2 cells was determined by cell invasion assay.

**Results:**

Stat3 was activated by IL-6 in Capan-2 cells; protein expression of p-Stat3 was increased significantly in Capan-2 cells. IL-6 remarkably promoted the growth of Capan-2 cells (*P *< 0.05), and VEGF and MMP-2 mRNA and protein expression were increased significantly. Also, IL-6 increased the invasion ability of Capan-2 cells. AG490 inhibited Stat3 activation in SW1990 cells. Western blotting and immunocytochemistry analysis showed that p-Stat3 protein expression was decreased significantly with AG490 treatment in SW1990 cells. AG490 remarkably inhibited the growth of Capan-2 cells (*P *< 0.05), and VEGF and MMP-2 mRNA and protein expression was decreased significantly. And AG490 decreased the invasion ability of SW1990 cells.

**Conclusions:**

Abnormal activation of Stat3 plays an important role in the invasion and metastasis of pancreatic cancer. Activation and blocking of the Stat3 signaling pathway can affect invasion ability and expression of the VEGF and MMP-2 genes in pancreatic cancer cells. The Stat3 signaling pathway may provide a novel therapeutic target for treatment of pancreatic cancer.

## Introduction

Pancreatic cancer is one of the most virulent malignances, with an overall 5-year survival rate of only 3-5% and a median survival time after diagnosis of less than 6 months[1]. This highly lethal disease is usually diagnosed in an advanced stage, when there are few or no effective therapies[2]. Even among patients undergoing a potentially curative resection, the long-term outcome remains unsatisfactory because of early recurrence and metastatic disease[3].

Despite the immensity of the clinical problem, the biology of pancreatic cancer remains only poorly understood. Signal transducer and activator of transcription (Stat) proteins were initially described in the context of regulating physiological cell signaling. An increasing number of studies have implicated Stat protein activation, particularly Stat3, in transformation and tumor progression[4]. Activated Stat3 has been shown to promote cell proliferation, metastasis, and angiogenesis, as well as protect tumor cells from apoptosis by regulating associated genes, such as Bcl-xL, Mcl-1, Bcl-2, Fas, cyclin D1, survivin, c-Myc, VEGF, MMP-2, and MMP-9[5-7].

Recently, accumulating evidence has indicated that abnormalities in the Stat3 pathway are involved in the oncogenesis of several cancers. For example, Scholz [8] and coworkers reported that activation of the Stat3 signaling pathway plays an important role in the progression of pancreatic cancer, and constitutive activation of Stat3 correlates with cell proliferation in stomach adenocarcinoma[9], prostate cancer[10], breast carcinoma[11], and non-small cell lung cancer[12] and also inhibits apoptosis[13,14]. Conversely, inhibition of the Stat pathway suppresses cancer cell growth and invasion and induces apoptosis in various cancers[8,11,15,16].

Jak is responsible for the tyrosine phosphorylation of Stat3 in response to extracellular signals and oncogenes. The newly described Jak inhibitor AG490 blocks the constitutive activation of Stat3[17]. AG490 was used to selectively block the Jak/Stat3 signaling pathway and inhibit activation of Stat3 in colorectal cancer cells[18].

The pleiotropic cytokine interleukin-6 (IL-6) is a major activator of Stat3; IL-6 stimulates the formation of tyrosine-phosphorylated Stat3 (p-Stat3) in cancer cells[19,20]. Through the Jak/Stat3 signaling pathway, IL-6 plays an important role in cell proliferation, apoptosis, metastasis, and other biological activities [21].

In the present study, we used AG490 to deplete Stat3 protein in the human pancreatic cancer cell line SW1990 and IL-6 to activate Stat3 protein in the human pancreatic cancer cell line Capan-2; we then investigated the changes in cell proliferation and invasion. We also examined the expression of Stat3 and its active phosphorylated form in human pancreatic cancer cell lines. In addition, we evaluated the changes in matrix metalloproteinase 2 (MMP-2) and vascular endothelial growth factor (VEGF) mRNA and protein expression. Our aim was to demonstrate that the Stat3 signaling pathway may be critical for the invasive behavior of pancreatic tumors. Inhibition of this pathway may offer a novel strategy for pancreatic cancer treatment.

## Methods

### Cells and reagents

The human pancreatic cancer cell lines SW1990 and Capan-2 were obtained from the American Type Culture Collection. Tumor cells were maintained in Dulbecco's modified Eagle's medium (DMEM), supplemented with 10% fetal calf serum, 100 units/ml penicillin and 100 μg/mL streptomycin, in a humidified incubator with an atmosphere of 5% CO_2 _and 95% air at 37°C. AG490 (Sigma, St Louis, MO, USA) was dissolved in ethanol, 5 mg/ml, and then diluted with the culture medium for experiments. SW1990 cells were treated with 20 μM AG490 for 24 hours. Recombinant IL-6 (Peprotech, Princeton, NJ, USA) was dissolved in 5-10 mmol/L acetic acid to a concentration of 0.1-0.5 mg/ml and then diluted with the culture medium for experiments. Capan-2 cells were treated with 100 ng/mL IL-6 for 24 hours.

### MTT assay

Cell viability was determined by 3-(4,5-dimethylthiazole-2-yl)-2.5-diphenyltetrazolium bromide (MTT) assay. Pancreatic cancer cells were seeded in 96-well culture plates in culture medium. After 24 hours, the medium was changed to fresh culture medium containing either 20 μM/L AG490 or 100 ng/ml IL-6. MTT assays were performed 24, 48, and 72 hours after AG490 and IL-6 treatment. At the time of the assay, the cells were stained with 20 μL MTT (5 mg/ml) (Sigma, St Louis, MO, USA) at 37°C for 4 hours and subsequently made soluble in 150 μL of DMSO. Absorbance was measured at 490 nm using a microtiter plate reader (Wako, Osaka, Japan). The results were used to obtain cell growth curves.

### Quantification by real-time PCR

Total RNA was isolated using TRIzol LS (Invitrogen, Carlsbad, CA, USA). The concentration and purity of RNA was determined using a spectrophotometer. cDNA was synthesized with M-MLV reverse transcriptase (Promega, Madison, WI, USA). Quantitative real-time polymerase chain reaction (RT-PCR) assays were carried out using SYBR Green Real-Time PCR Master Mix (Toyobo, Osaka, Japan) and realplex S RT-PCR amplification equipment (Eppendorf, Hamburg, Germany).

The primers and amplicon sizes were as follows: MMP-2 sense strand 5'-TAG CAT GTC CCT ACC GAG TCT-3', antisense strand 5'- ATT GGA TGG CAG TAG CTG C-3', with a product length of 151 bp; VEGF sense strand 5'-CTG TCT TGG GTG CAT TGG A-3', antisense strand 5'-ATT GGA TGG CAG TAG CTG C-3', with a product length of 152 bp; β-actin sense strand 5'-CAC CAA CTG GGA CGA CAT-3', antisense strand 5'-ATC TGG GTC ATC TTC TCG C-3', with a product length of 138 bp (Shenggong Biotech, Shanghai, China).

PCR parameters were as follows: 95°C for 5 minutes, then 95°C for 30 seconds, 56°C for 30 seconds, 72°C for 40 seconds for 40 cycles. A standard calibration curve for expression of each mRNA was generated using 8-fold dilutions of a control RNA sample. MMP-2 and VEGF mRNA expression was calculated as a ratio to that of β-actin.

### Immunocytochemistry

SW1990 cells and Capan-2 cells were grown on poly-L-lysine-coated slides in a 6-well plate; after treatment with AG490 and IL-6, respectively, the slides of 4 groups were washed twice with PBS and fixed in 4% paraformaldehyde for 30 minutes at room temperature. Immunostaining was performed using the streptavidin-biotin complex method with the UltraSensitive S-P Kit (Fuzhou Maxim Biotech, Fuzhou, China). The slides were pretreated first with 0.3% hydrogen peroxide in PBS for 10 minutes to inactivate endogenous peroxidase, and then microwave antigen retrieval was performed with 0.01 mol/L citrate buffer at pH 6.0 for 20 minutes, followed by incubation with rabbit anti-human Phospho-Tyr705-Stat3 polyclonal antibody (1:100, Cell Signaling Technology, Danvers, MA, USA) at 4°C overnight.

For negative controls, slides were processed as above but treated with PBS, instead of the primary antibody/biotinylated secondary antibody, for 30 minutes and peroxidase-labeled streptavidin for 30 minutes. Color reaction was developed with 3, 3'-diaminobenzidine as a chromogen.

Finally, the slides were counterstained with hematoxylin, dehydrated through graded alcohol, and observed under the microscope. We used the Image Analysis System for protein analysis; 5 different views were selected for each slide (400 times). Integrated optical density was used as the measurement of staining strength.

### Western blotting

Whole-cell protein extracts and nuclear protein extracts from pancreatic cancer cells were prepared with RIPA Lysis Buffer (Santa Cruz Biotechnology, Santa Cruz, CA, USA) and Nuclear Extract Kit (Active Motif, Carlsbad, CA, USA), respectively, according to the manufacturers' instructions. Protein concentrations were determined using an assay kit (Bio-Rad, Hercules, CA, USA).

Lysates containing 100 μg of protein were mixed with loading buffer with 5% β-mercaptoethanol and heated for 5 minutes at 100°C. Samples were separated by sodium dodecyl sulfate-polyacrylamide gel electrophoresis and transferred onto nitrocellulose membranes by semi-dry blotting.

Membranes were incubated in blocking buffer (tris-buffered saline [TBS], 0.1% Tween 20, and 5% non-fat dry milk) for 1 hour at room temperature, followed by hybridization with anti-p-Stat3 (tyr-705) antibody (Cell Signaling Technology, 1:1000 dilution), anti-Stat3 antibody (Cell Signaling Technology, 1:1000 dilution), anti-MMP-2 antibody (Santa Cruz Biotechnology, 1:500 dilution), anti-VEGF antibody (Santa Cruz Biotechnology, 1:500 dilution) or anti β-actin antibody (Lab Vision, Fremont, CA, USA, 1:100 dilution) at 4°C overnight.

After 3 washes in TBS/0.1% Tween 20, the membranes underwent hybridization with a horseradish peroxidase-conjugated secondary antibody rabbit IgG (Santa Cruz Biotechnology, 1:5000 dilution) for 1 hour at room temperature. After 3 washes in TBS/0.1% Tween 20, signals were detected by chemiluminescence using western blotting luminol reagent (Santa Cruz Biotechnology).

### Invasion assay

The invasion assay was performed using a specialized invasion chamber (Chemicon, Temecula, CA, USA). The inserts contained an 8-μm pore size polycarbonate membrane with a precoated thin layer of basement membrane matrix (ECMatrix). Briefly, media supplemented with 10% fetal bovine serum was poured into the lower chamber as a hemo-attractant. After reaching 60-70% subconfluence, pancreatic cancer cells were trypsinized and resuspended in DMEM (1×10^6 ^cells/ml), and 0.3 ml was re-seeded into the upper chambers. Cells were cultured in medium containing either vehicle alone (control) or indicated doses of AG490.

After 24 hours of incubation at 37°C, non-invasive cells were removed from the upper surface of the membrane using a moist cotton-tipped swab. Invasive cells on the lower surface of the membrane, which had invaded the ECMatrix and had migrated through the polycarbonate membrane, were stained with the staining solution for 20 minutes and rinsed with distilled water several times. Invasiveness was quantitated by selecting 10 different views (400 times) and counting the number of invasion cells.

### Statistical analysis

All assays were conducted 3 times and found to be reproducible. Data were expressed as mean ± SD. Statistical correlation of data between groups was checked for significance by Student's *t *test. Differences with *P *< 0.05 were considered significant. These analyses were performed using SPSS 11.0 software.

## Results

### Effects of AG490 and IL-6 on growth in pancreatic cancer cells

Because Stat3 activation was positively associated with proliferation potential in cancer cells, we measured the absorbance of the SW1990 cell line in the presence of AG490. Incubation with 20 μM/L AG490 for 72 hours markedly reduced proliferation of SW1990 cells (*P *< 0.05), but incubation with 20 μM/L AG490 for 24 and 48 hours did not reduce proliferation of SW1990 cells (*P *> 0.05). We measured the absorbance of the Capan-2 cell line in the presence of IL-6, a cytokine that can active the Jak/Stat3 signaling pathway. Incubation with 100 ng/ml IL-6 for 48 and 72 hours increased proliferation of Capan-2 cells significantly (*P *< 0.05) , but incubation with 100 ng/ml IL-6 for for 24 hours did not increase proliferation of SW1990 cells (*P *> 0.05).

**Figure 1:**
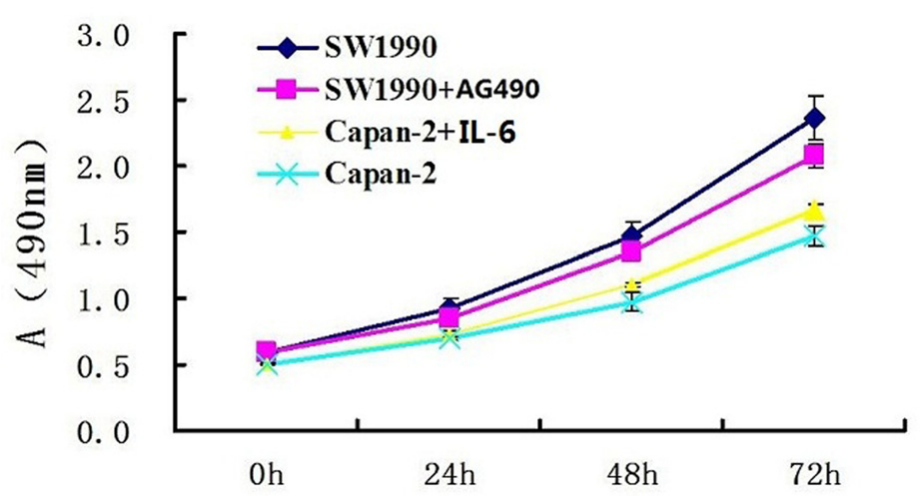
**Pancreatic cancer cell growth was detected by MTT assay**. SW1990 and Capan-2 cells growing in 96-well plates were treated with AG490 and interleukin-6 (IL-6), respectively, for 24, 48 and 72 hours. Incubation with 20 μM/L AG490 for 72 hours markedly reduced proliferation of SW1990 cells (*P *= 0.000), but incubation with 20 μM/L AG490 for 24, 48 hours did not reduce proliferation of SW1990 cells (*P *= 0.051, *P *= 0.060). Incubation with 100 ng/ml IL-6 for 48 and 72 hours increased proliferation of Capan-2 cells significantly (*P *= 0.001, *P *= 0.000) , but incubation with 100 ng/ml IL-6 for for 24 hours did not increase proliferation of SW1990 cells (P = 0.073). Data are mean ± SD of 8 wells. A = Absorbance.

Because of these results, cell invasion assay was performed with doses of 20 μM/L AG490 for 24 hours and 100 ng/ml IL-6 for for 24 hours to ignore the influence of cell viability. The growth curve was obtained according to the absorbance of the cells. (Figure 1)

### Effects of AG490 and IL-6 on VEGF and MMP-2 mRNA expression in pancreatic cancer cells

**Figure 2:**
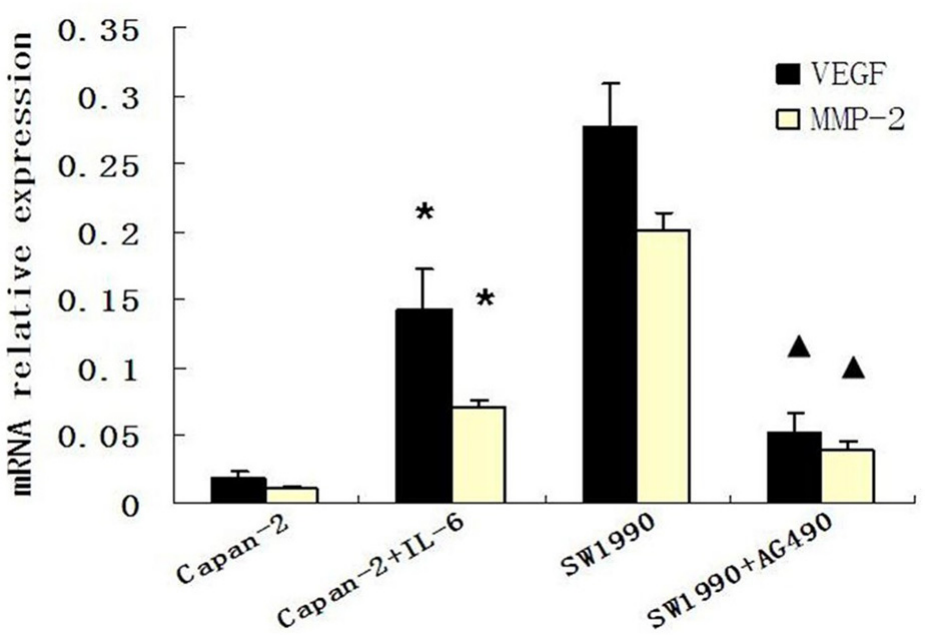
**VEGF and MMP-2 mRNA levels in SW1990 and Capan-2 cells were detected by real time PCR**. The extracted total RNA was reverse-transcribed into single-stranded cDNA, and real-time PCR was performed. Interleukin-6 (IL-6) markedly increased MMP-2 and VEGF mRNA expression in Capan-2 cells(*P *= 0.000, *P *= 0.000). AG490 significantly decreased MMP-2 and VEGF mRNA expression in SW1990 cells(*P *= 0.008, *P *= 0.000). β-actin was used as an endogenous control. * *P *< 0.01, versus Capan-2 cell group; #*P *< 0.01, versus SW1990 cell group.

The mRNA levels of the VEGF and MMP-2 genes in SW1990 and Capan-2 cells were examined by RT-PCR. RNA samples were extracted from SW1990 cells treated for 24 hours with 20 μM AG490 and then subjected to RT-PCR for MMP-2, VEGF and β-actin. AG490 significantly decreased the expression of MMP-2 and VEGF mRNAs in SW1990 cells. Treatment with 100 ng/ml IL-6 in Capan-2 cells for 24 hours markedly increased the VEGF and MMP-2 mRNA levels. Data are presented as mean ± SD. (Figure 2)

### Effects of AG490 and IL-6 on p-Stat3 protein expression in pancreatic cancer cells

**Figure 3:**
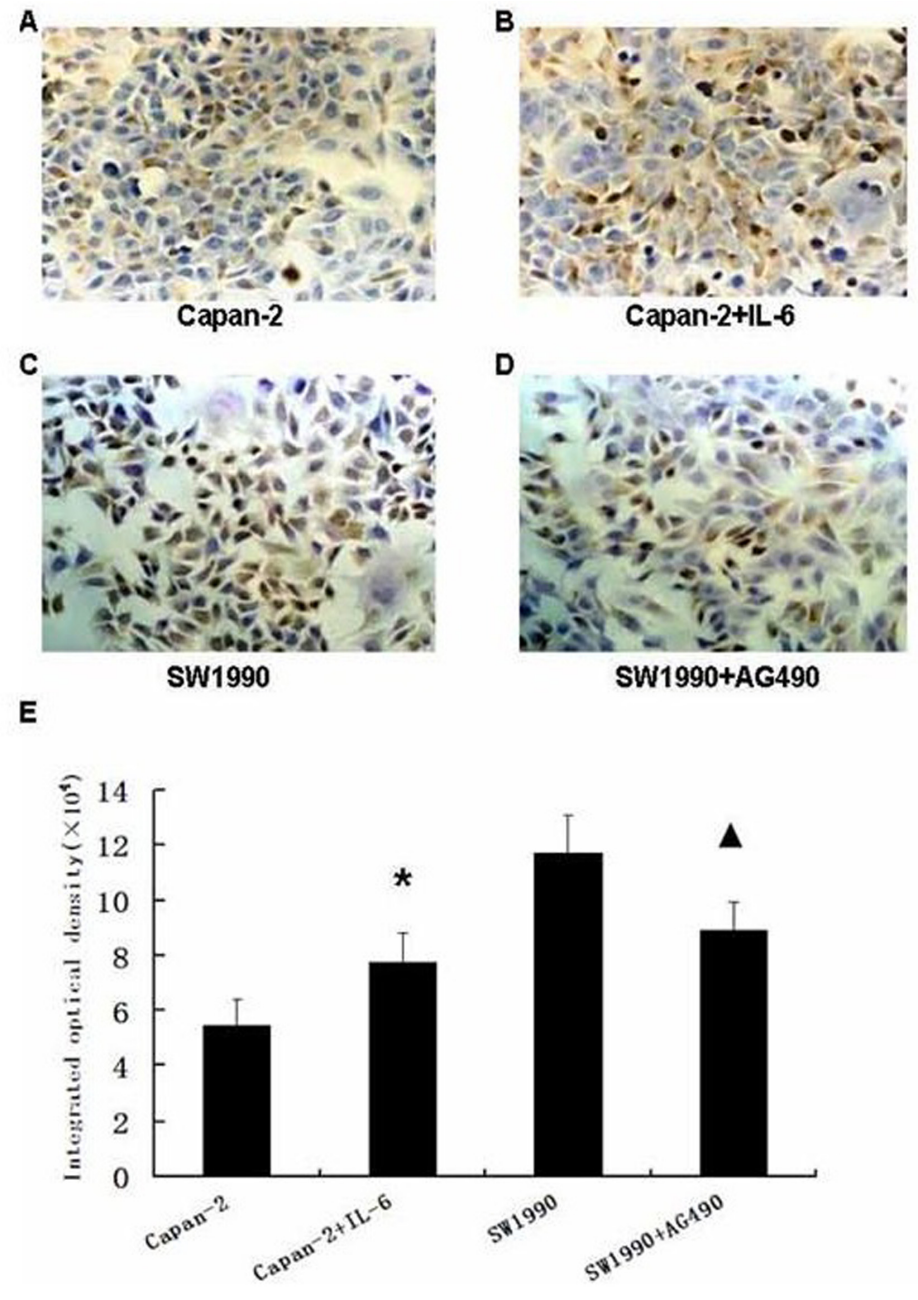
**p-Stat3 protein expression was detected by immunocytochemistry**. Immunocytochemical staining showed that p-Stat3 was mainly expressed in the nucleus and weakly expressed in the cytoplasm of SW990 cells and Capan-2 cells. Expression of p-Stat3 protein in Capan-2 cells (A) and SW1990 cells (C). After treatment with interleukin-6 (IL-6) for 24 hours on Capan-2 cells (B), we observed that the intensity of p-Stat3 expression increased(*P *= 0.012). After treatment with AG490 for 24 hours on SW1990 cells (D), we observed that the intensity of p-Stat3 expression decreased (*P *= 0.006) (original magnification, ×400). (E) Integrated optical density of every group. Bars indicate mean ± SD. * *P *< 0.01, versus Capan-2 cell group; # *P *< 0.01, versus SW1990 cell group.

Immunocytochemical staining showed that p-Stat3 was mainly expressed in the nucleus and weakly expressed in the cytoplasm of SW1990 and Capan-2 cells. Treatment with 20 μM/L AG490 in SW1990 cells for 24 hours markedly decreased the intensity of p-Stat3 expression. Treatment with 100 ng/ml IL-6 in Capan-2 cells for 24 hours significantly increased the intensity of p-Stat3 expression. (Figure 3)

### Effects of AG490 and IL-6 on p-Stat3, VEGF and MMP-2 protein levels in pancreatic cancer cells

**Figure 4:**
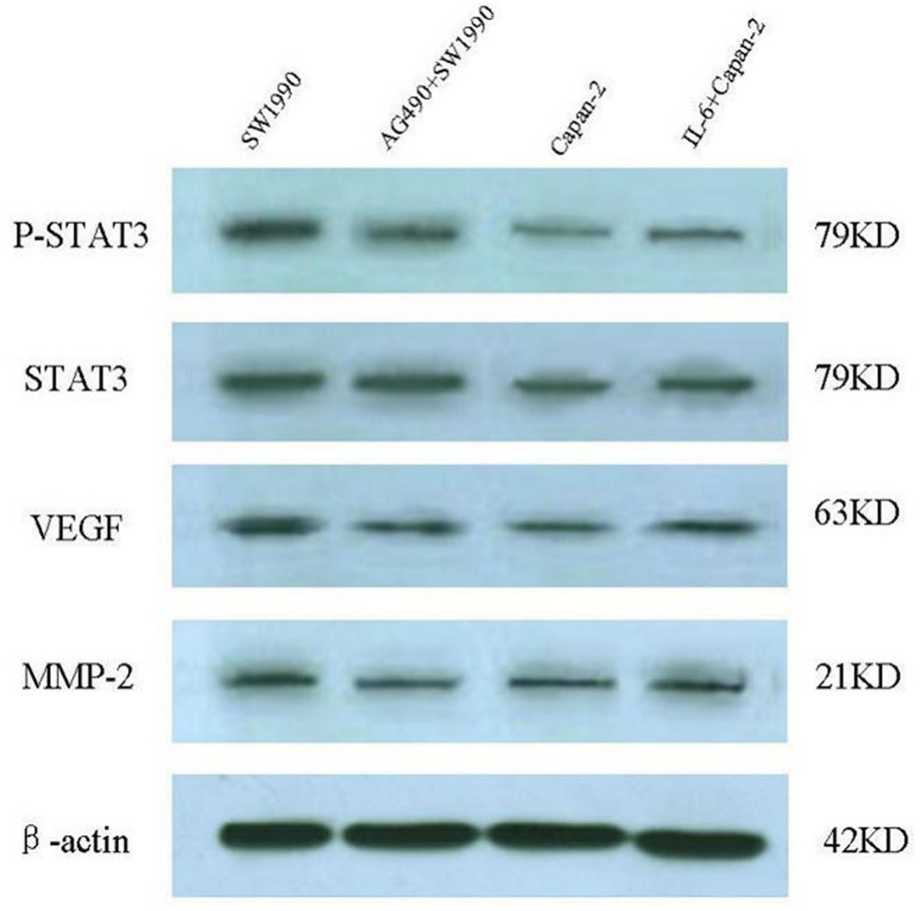
**Stat3, p-Stat3, MMP-2 and VEGF protein expression in SW1990 and Capan-2 cells were detected by Western blotting**. Protein samples extracted from SW1990 and Capan-2 cells treated for 24 hours with AG490 and interleukin-6 (IL-6), respectively, were subjected to western blotting for Stat3, p-Stat3, MMP-2, VEGF and β-actin proteins. AG490 and IL-6 did not affect total Stat3 protein levels. AG490 decreased p-Stat3, MMP-2 and VEGF protein expression in SW1990 cells(*P *= 0.010, *P *= 0.000, *P *= 0.009). IL-6 markedly increased p-Stat3, MMP-2 and VEGF protein expression in Capan-2 cells(*P *= 0.000, *P *= 0.011, *P *= 0.005). The levels of β-actin expression were determined as a control for equivalent protein loading.

We used western blotting to examine the effects of AG490 and IL-6 on p-Stat3, VEGF, and MMP-2 protein levels of SW1990 and Capan-2 cells. AG490 did not affect total Stat3 protein levels in SW1990 cells after treatment with 20 μM/L AG490 for 24 hours but did suppress p-Stat3, VEGF, and MMP-2 protein levels. Treatment of Capan-2 cells with 100 ng/ml IL-6 for 24 hours increased p-Stat3, VEGF, and MMP-2 protein expression levels significantly. (Figure 4)

### Effects of AG490 and IL-6 on invasive ability of pancreatic cancer cells

**Figure 5:**
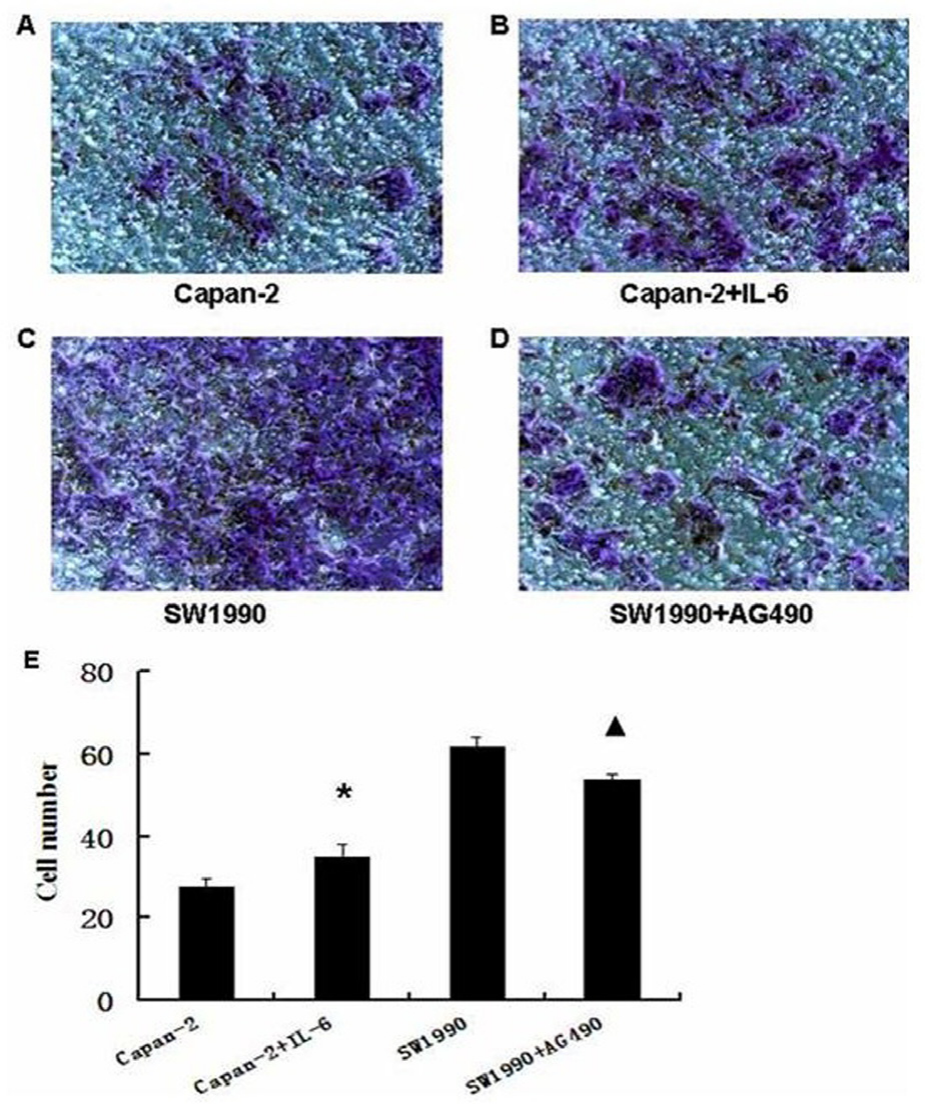
**The invasion assay was performed using a specialized invasion chamber**. The invasion chamber included a 24-well tissue-culture plate with 12 cell-culture inserts. The blue-stained cells are those that invaded the basement membrane matrix (ECMatrix) and migrated through the polycarbonate membrane to the lower surface of the membrane. The invasion assay indicated that interleukin-6 (IL-6) significantly increased the invasion ability of Capan-2 cells (A, B) (*P *= 0.004), and AG490 markedly reduced invasion of SW1990 cells (C, D) (*P *= 0.010) (original magnification ×200). (E) Effects of AG490 and IL-6 on invasion ability of pancreatic cancer cells. Bars indicate mean ± SD. * *P *< 0.05, versus Capan-2 cell group; #*P *< 0.01, versus SW1990 cell group.

To evaluate the effects of regulation of Stat3 activity on pancreatic cancer invasion, we performed an in vitro invasion assay using AG490 and IL-6 (Figure 5). According to the number of invasive cells, AG490 markedly reduced invasion of SW1990 cells (*P *< 0.05) compared with the vehicle-treated cells. IL-6 increased the invasion ability of Capan-2 cells significantly (*P *< 0.05). (Figure 5)

## Discussion

The Jak/Stat3 signaling pathway plays a vital role in regulating a number of pathways in tumorigenesis, including cell cycle progression, apoptosis, tumor angiogenesis, and tumor cell evasion of the immune system. Cytokines and growth factors bind to the membrane receptors that activate the nonreceptor tyrosine kinase. Once the tyrosine is phosphorylated, two Stat3 monomers form dimers through reciprocal phosphotyrosine-SH_2 _interactions, translocate to the nucleus, where they bind to Stat3-specific DNA-response elements of target genes, and induce gene transcription[22].

During malignant transformation, Stat3 frequently is overexpressed and constitutively activated by tyrosine phosphorylation. Previous studies have demonstrated that activated Stat3 is overexpressed in human pancreatic cancer tissues and cell lines[23].

Despite the clear importance of Stat3 in cell proliferation, invasion, metastasis, and survival in human pancreatic cancer, its potential molecular contribution to pancreatic cancer invasion and metastasis has not been fully characterized. In our previous studies, we compared the levels of p-Stat3 protein and the invasion ability between SW1990 and CaPan-2 cell lines. We found that p-Stat3 protein levels were significantly higher in SW1990 cells compared to the CaPan-2 cells. Furthermore, invasion assay in vitro indicated significant invasion ability of SW-1990 cells, while weak invasion ability was observed in CaPan-2 cells[24]. In the present study, we used immunocytochemistry and Western blotting to detect the level of p-Stat3 protein in SW1990 and Capan-2 cells after theatment with AG490 or IL-6. We found that the p-Stat3 protein level was significantly decreased in SW1990 cells after treatment with AG490 and markedly increased in Capan-2 cells after treatment with IL-6. These results demonstrate that AG490 strongly suppresses Stat3 activity and that IL-6 promotes Stat3 activity in pancreatic cancer cell lines.

Stat3 is an oncogene that is constitutively active in many tumor types and promotes cell proliferation and survival[21,25]. Inappropriate and constitutive activation of Stat3 may be responsible for pancreatic cancer progression by regulating the expression of target genes, such as c-Myc, Bcl-xL, p21WAF1, and cyclinD1, and functional inactivation of Stat3 by dominant-negative Stat3 or AG490 could inhibit the proliferation and promote the apoptosis of pancreatic cancer cells[8,26].

Moreover, evidence indicates that constitutive activation of Stat3 influences invasion and metastasis. For example, activation of Stat3 in thymic epithelial tumors[27], colorectal adenocarcinoma[28], and cutaneous squamous cell carcinoma[29] correlates with invasion and lymph node metastasis.

In our study, we examined the effects of AG490 and IL-6 on growth capability of pancreatic cancer cells. The MTT assay indicated that IL-6 can stimulate the growth of Capan-2 cells, and proliferation of SW1990 cells was attenuated when cells were treated with AG490. We examined the invasive ability of these cells using a cell invasion assay kit. We found that SW1990 cells showed a weaker level of invasion after treatment with AG490. In contrast, Capan-2 cell invasion was significantly increased by IL-6. Therefore, there is a strong relationship between Stat3 activity and the invasive ability of human pancreatic cancer cells.

Tumor invasion and metastasis depend on angiogenesis, which is the formation of new blood vessels from a pre-existing network of capillaries. VEGF is known to be a potent angiogenic mitogen that plays an important role in tumor angiogenesis, invasion, and metastasis[30]. The role of Stat3 in angiogenesis was first shown when VEGF was found to be a direct target of Stat3 in mouse melanoma cells[6] and then confirmed by a study in a human pancreatic cancer system[31].

A recent study has reported that constitutively activated Stat3 directly activated the VEGF promoter, whereas dominant-negative Stat3 inhibited the VEGF promoter. Furthermor, a Stat3-responsive element on the VEGF promoter was identified using a protein-DNA binding assay and confirmed using a promoter mutagenesis assay[31]. Our previous study also found that silencing of the Stat3 gene by RNAi decreases VEGF expression in the pancreatic cancer cell line SW1990[^23^]. In the present study, we also found that AG490 significantly decreased the mRNA and protein expression of VEGF in SW1990 cells, and IL-6 markedly increased the VEGF mRNA and protein expression in Capan-2 cells.

According to studies that have used clinical samples of pancreatic cancer and pancreatic cancer cell lines, MMPs play important roles in tumor cell invasion and metastasis by degrading components of the basement membranes and extracellular matrix[32,33]. Specifically, activated Stat3 regulates tumor invasion of melanoma cells by regulating the gene transcription of MMP-2. Furthermore, a high-affinity Stat3-binding element is identified in the MMP-2 promoter and Stat3 could upregulate the transcription of MMP-2 through direct interaction with the MMP-2 promoter[7,34]. In our present study, the use of AG490 markedly reduced MMP-2 mRNA and protein expression in SW1990 cells, and IL-6 significantly increased MMP-2 mRNA and protein expression in Capan-2 cells through activation of the Stat3 signaling pathway.

Collectively, our findings strongly suggest that the Jak/Stat3 pathway plays a significant role in pancreatic cancer cell invasion. Targeting of Stat3 activation may prove to be a more effective approach to controlling invasion than merely targeting individual molecules, such as VEGF and MMP-2, possibly representing a novel approach to regulating pancreatic cancer invasion.

## Competing interests

The authors declare that they have no competing interests.

## Authors' contributions

QZJ supervised the design of the experiments and analysed and interpreted of data. HC carried out the MTT assay and the invasion assay, conceived the study and drafted the manuscript. YG was involved in Western-blotting, real-time PCR, drafting of the manuscript and design of the study. JT carried out the immunocytochemistry studies. HKJ and CJ participated in the design and coordination of the work involved. All authors read and approved the final manuscript.

## References

[B1] Jemal A Siegel R Ward E Murray T Xu J Thun MJ Cancer statistics, 2007 CA Cancer J Clin 2007 57 43 66 10.3322/canjclin.57.1.43 17237035

[B2] Postier RG The challenge of pancreatic cancer Am J Surg 2003 186 579 582 10.1016/j.amjsurg.2003.08.018 14672761

[B3] Neoptolemos JP Cunningham D Friess H Bassi C Stocken DD Tait DM Adjuvant therapy in pancreatic cancer: historical and current perspectives Ann Oncol 2003 14 675 692 10.1093/annonc/mdg207 12702520

[B4] Bromberg J Darnell JE Jr The role of STATs in transcriptional control and their impact on cellular function Oncogene 2000 19 2468 2473 10.1038/sj.onc.1203476 10851045

[B5] Huang S Regulation of metastases by signal transducer and activator of transcription 3 signaling pathway: clinical implications Clin Cancer Res 2007 13 1362 1366 10.1158/1078-0432.CCR-06-2313 17332277

[B6] Niu G Wright KL Huang M Song L Haura E Turkson J Constitutive Stat3 activity up-regulates VEGF expression and tumor angiogenesis Oncogene 2002 21 2000 2008 10.1038/sj.onc.1205260 11960372

[B7] Xie TX Wei D Liu M Gao AC Ali-Osman F Sawaya R Stat3 activation regulates the expression of matrix metalloproteinase-2 and tumor invasion and metastasis Oncogene 2004 23 3550 3560 10.1038/sj.onc.1207383 15116091

[B8] Scholz A Heinze S Detjen KM Peters M Welzel M Hauff P Activated signal transducer and activator of transcription 3 (STAT3) supports the malignant phenotype of human pancreatic cancer Gastroenterology 2003 125 891 905 10.1016/S0016-5085(03)01064-3 12949733

[B9] Yu LF Cheng Y Qiao MM Zhang YP Wu YL Activation of STAT3 signaling in human stomach adenocarcinoma drug-resistant cell line and its relationship with expression of vascular endothelial growth factor World J Gastroenterol 2005 11 875 879 15682485 10.3748/wjg.v11.i6.875 PMC4250601

[B10] Lee SO Lou W Qureshi KM Mehraein-Ghomi F Trump DL Gao AC RNA interference targeting Stat3 inhibits growth and induces apoptosis of human prostate cancer cells Prostate 2004 60 303 309 10.1002/pros.20072 15264241

[B11] Zhang F Li C Halfter H Liu J Delineating an oncostatin M-activated STAT3 signaling pathway that coordinates the expression of genes involved in cell cycle regulation and extracellular matrix deposition of MCF-7 cells Oncogene 2003 22 894 905 10.1038/sj.onc.1206158 12584569

[B12] Alvarez JV Greulich H Sellers WR Meyerson M Frank DA Signal transducer and activator of transcription 3 is required for the oncogenic effects of non-small-cell lung cancer-associated mutations of the epidermal growth factor receptor Cancer Res 2006 66 3162 3168 10.1158/0008-5472.CAN-05-3757 16540667

[B13] Shen Y Devgan G Darnell JE Jr Bromberg JF Constitutively activated Stat3 protects fibroblasts from serum withdrawal and UV-induced apoptosis and antagonizes the proapoptotic effects of activated Stat1 Proc Natl Acad Sci USA 2001 98 1543 1548 10.1073/pnas.041588198 11171987 PMC29293

[B14] Zamo A Chiarle R Piva R Howes J Fan Y Chilosi M Anaplastic lymphoma kinase (ALK) activates Stat3 and protects hematopoietic cells from cell death Oncogene 2002 21 1038 1047 10.1038/sj.onc.1205152 11850821

[B15] Blaskovich MA Sun J Cantor A Turkson J Jove R Sebti SM Discovery of JSI-124 (cucurbitacin I), a selective Janus kinase/signal transducer and activator of transcription 3 signaling pathway inhibitor with potent antitumor activity against human and murine cancer cells in mice Cancer Res 2003 63 1270 1279 12649187

[B16] Mora LB Buettner R Seigne J Diaz J Ahmad N Garcia R Constitutive activation of Stat3 in human prostate tumors and cell lines: direct inhibition of Stat3 signaling induces apoptosis of prostate cancer cells Cancer Res 2002 62 6659 6666 12438264

[B17] Meydan N Grunberger T Dadi H Shahar M Arpaia E Lapidot Z Inhibition of acute lymphoblastic leukaemia by a Jak-2 inhibitor Nature 1996 379 645 648 10.1038/379645a0 8628398

[B18] Xiong H Zhang ZG Tian XQ Sun DF Liang QC Zhang YJ Inhibition of JAK1, 2/STAT3 signaling induces apoptosis, cell cycle arrest, and reduces tumor cell invasion in colorectal cancer cells Neoplasia 2008 10 287 297 18320073 10.1593/neo.07971 PMC2259457

[B19] Yang J Liao X Agarwal MK Barnes L Auron PE Stark GR Unphosphorylated STAT3 accumulates in response to IL-6 and activates transcription by binding to NFkappaB Genes Dev 2007 21 1396 1408 10.1101/gad.1553707 17510282 PMC1877751

[B20] Sekikawa A Fukui H Fujii S Ichikawa K Tomita S Imura J REG Ialpha protein mediates an anti-apoptotic effect of STAT3 signaling in gastric cancer cells Carcinogenesis 2008 29 76 83 10.1093/carcin/bgm250 18024479

[B21] Hodge DR Hurt EM Farrar WL The role of IL-6 and STAT3 in inflammation and cancer Eur J Cancer 2005 41 2502 2512 10.1016/j.ejca.2005.08.016 16199153

[B22] Bowman T Garcia R Turkson J Jove R STATs in oncogenesis Oncogene 2000 19 2474 2488 10.1038/sj.onc.1203527 10851046

[B23] Qiu Z Huang C Sun J Qiu W Zhang J Li H RNA interference-mediated signal transducers and activators of transcription 3 gene silencing inhibits invasion and metastasis of human pancreatic cancer cells Cancer Sci 2007 98 1099 1106 10.1111/j.1349-7006.2007.00485.x 17459060 PMC11158338

[B24] Huang C Cao J Huang KJ Zhang F Jiang T Zhu L Inhibition of STAT3 activity with AG490 decreases the invasion of human pancreatic cancer cells in vitro Cancer Sci 2006 97 1417 1423 10.1111/j.1349-7006.2006.00340.x 17054436 PMC11159136

[B25] Haura EB Turkson J Jove R Mechanisms of disease: Insights into the emerging role of signal transducers and activators of transcription in cancer Nat Clin Pract Oncol 2005 2 315 324 10.1038/ncponc0195 16264989

[B26] Toyonaga T Nakano K Nagano M Zhao G Yamaguchi K Kuroki S Blockade of constitutively activated Janus kinase/signal transducer and activator of transcription-3 pathway inhibits growth of human pancreatic cancer Cancer Lett 2003 201 107 116 10.1016/S0304-3835(03)00482-8 14580692

[B27] Chang KC Wu MH Jones D Chen FF Tseng YL Activation of STAT3 in thymic epithelial tumours correlates with tumour type and clinical behaviour J Pathol 2006 210 224 233 10.1002/path.2041 16917804

[B28] Kusaba T Nakayama T Yamazumi K Yakata Y Yoshizaki A Nagayasu T Expression of p-STAT3 in human colorectal adenocarcinoma and adenoma; correlation with clinicopathological factors J Clin Pathol 2005 58 833 838 10.1136/jcp.2004.023416 16049285 PMC1770863

[B29] Suiqing C Min Z Lirong C Overexpression of phosphorylated-STAT3 correlated with the invasion and metastasis of cutaneous squamous cell carcinoma J Dermatol 2005 32 354 360 16043897 10.1111/j.1346-8138.2005.tb00906.x

[B30] Grunstein J Roberts WG Mathieu-Costello O Hanahan D Johnson RS Tumor-derived expression of vascular endothelial growth factor is a critical factor in tumor expansion and vascular function Cancer Res 1999 59 1592 1598 10197634

[B31] Wei D Le X Zheng L Wang L Frey JA Gao AC Stat3 activation regulates the expression of vascular endothelial growth factor and human pancreatic cancer angiogenesis and metastasis Oncogene 2003 22 319 329 10.1038/sj.onc.1206122 12545153

[B32] Matsuyama Y Takao S Aikou T Comparison of matrix metalloproteinase expression between primary tumors with or without liver metastasis in pancreatic and colorectal carcinomas J Surg Oncol 2002 80 105 110 10.1002/jso.10106 12173379

[B33] Tan X Egami H Ishikawa S Sugita H Kamohara H Nakagawa M Involvement of matrix metalloproteinase-7 in invasion-metastasis through induction of cell dissociation in pancreatic cancer Int J Oncol 2005 26 1283 1289 15809719

[B34] Xie TX Huang FJ Aldape KD Kang SH Liu M Gershenwald JE Activation of stat3 in human melanoma promotes brain metastasis Cancer Res 2006 66 3188 3196 10.1158/0008-5472.CAN-05-2674 16540670

